# Comparison between xenogeneic and allogeneic adipose mesenchymal stem cells in the treatment of acute cerebral infarct: proof of concept in rats

**DOI:** 10.1186/s12967-015-0406-3

**Published:** 2015-02-01

**Authors:** María Gutiérrez-Fernández, Berta Rodríguez-Frutos, Jaime Ramos-Cejudo, Laura Otero-Ortega, Blanca Fuentes, María Teresa Vallejo-Cremades, Borja Enrique Sanz-Cuesta, Exuperio Díez-Tejedor

**Affiliations:** Department of Neurology and Stroke Centre, Neuroscience and Cerebrovascular Research Laboratory, La Paz University Hospital, Neuroscience Area of IdiPAZ (Health Research Institute), Autónoma University of Madrid, Paseo de la Castellana 261, 28046 Madrid, Spain

**Keywords:** Allogeneic and xenogeneic AD-MSCs, Functional recovery, Safety, Stroke

## Abstract

**Background:**

Rat adipose tissue-derived-mesenchymal stem cells (rAD-MSCs) have proven to be safe in experimental animal models of stroke. However, in order to use human AD-MSCs (hAD-MSCs) as a treatment for stroke patients, a proof of concept is needed. We analyzed whether the xenogeneic hAD-MSCs were as safe and effective as allogeneic rAD-MSCs in permanent Middle Cerebral Artery Occlusion (pMCAO) in rats.

**Methods:**

Sprague–Dawley rats were randomly divided into three groups, which were intravenously injected with xenogeneic hAD-MSCs (2 × 10^6^), allogeneic rAD-MSCs (2 × 10^6^) or saline (control) at 30 min after pMCAO. Behavior, cell implantation, lesion size and cell death were evaluated. Brain markers such as GFAP (glial fibrillary acid protein), VEGF (vascular endothelial growth factor) and SYP (synaptophysin) and tumor formation were analyzed.

**Results:**

Compared to controls, recovery was significantly better at 24 h and continued to be so at 14 d after IV administration of either hAD-MSCs or rAD-MSCs. No reduction in lesion size or migration/implantation of cells in the damaged brain were observed in the treatment groups. Nevertheless, cell death was significantly reduced with respect to the control group in both treatment groups. VEGF and SYP levels were significantly higher, while those of GFAP were lower in the treated groups. At three months, there was no tumor formation.

**Conclusions:**

hAD-MSCs and rAD-MSCs were safe and without side effects or tumor formation. Both treatment groups showed equal efficacy in terms of functional recovery and decreased ischemic brain damage (cell death and glial scarring) and resulted in higher angiogenesis and synaptogenesis marker levels.

## Introduction

Worldwide, stroke is the second cause of death and the leading cause of disability [1]. Currently, only one thrombolytic agent, tissue plasminogen activator (tPA), is approved for the treatment of ischemic stroke; however, given its narrow therapeutic window, only 2–3% of all stroke patients can benefit from the use of tPA. Stroke is a clinical entity that requires more innovative treatments for both brain protection and repair. Cell-based therapy is an interesting area for developing a new approach to cerebral infarct.

Along these lines, adipose tissue-derived mesenchymal stem cells (AD-MSCs) from rodents have demonstrated safety, feasibility and efficacy in experimental animal models of stroke [2-4]. Moreover, AD-MSCs are abundant, easy to obtain with minimally invasive techniques and can be administrated without ethical concerns. In fact, rat AD-MSCs (rAD-MSCs) are as effective as rat bone marrow-derived MSCs (rBM-MSCs) following ischemic stroke in obtaining improved functional recovery in rats [2] and are even more effective in trophic factor secretion [3,5]. However, to test the safety of human AD-MSCs (hAD-MSCs) before using them as a treatment for stroke patients, a proof of concept is needed. Having demonstrated that rAD-MSCs are effective in experimental animals, it is now important to test whether xenogeneic hAD-MSCs are as safe and effective as allogeneic rAD-MSCs after stroke in rats.

We administered both hAD-MSCs (xenogeneic administration) and rAD-MSCs (allogeneic administration) in an experimental animal model of stroke to analyze and compare their safety and effectiveness.

## Methods

### Animals

The subjects were adult male Sprague–Dawley rats, with an average body weight range of 250–320 gr (Harlan Iberica S.L.). The animals were housed with free access to food and water at a room temperature of 21 ± 2°C, relative humidity of 45 ± 15% and a light/dark cycle of 12h (7:00 to 19:00). The procedure was carried out at our Cerebrovascular and Neuroscience Research Laboratory. All experiments were designed to minimize animal suffering in compliance with our medical school’s Ethical Committee for the Care and Use of Animals in Research (EU directives 86/609/CEE and 2003/65/CE). The researchers responsible for functional evaluation and the molecular and histological studies were blinded to the treatment groups.

### Study design

#### Isolation and characterization of AD-MSCs

The hAD-MSCs were provided by Cellerix and were obtained from lipoaspirates from healthy donors. The rAD-MSCs were extracted from abdominal adipose tissue of adult Sprague–Dawley rats (250 to 300 g) as previously described [2]. Briefly, the extracted tissue was washed with sterile PBS and digested with an equal volume of 0.075% type I collagenase (Sigma-Aldrich). The filtered cells were centrifuged at 390g for 10min and contaminating erythrocytes were removed to isolate the stromal vascular fraction (SVF) that was cultured in Dulbecco modified Eagle medium (1X) (DMEM Glu/Pyr, Gibco), 75 μl penicillin/streptomycin (Sigma-Aldrich) and 20% fetal bovine serum (PAA Laboratories). On the third pass, cells were trypsinized and counted before being administered to the experimental animals.

To confirm the presence or absence of MSCs surface markers using the flow cytometric technique, the cells were incubated for 20 min at 4°C in the dark with the following antibodies: CD90-fluorescein isothiocyanate (FITC) (AbD Serotec), CD29-Phycoerythrin (PE) (AbD Serotec), CD45-PE (AbD Serotec) and CD11b-PE (AbD Serotec). Matched isotype controls were purchased from Biolegend. At least 1×10^4^ cells per sample were acquired and analyzed [2].

#### Experimental groups

We operated on 63 rats and lost 6. Severe ischemic brain damage was the cause of death after induction of cerebral ischemia in 2 rats, hypothermia due to anesthesia in Magnetic Resonance Imaging (MRI) occurred in 3 rats and microembolization after allogeneic cell administration (rAD-MSCs) occurred in 1 rat.

The animals were randomly assigned to one of five experimental groups: 1-The Healthy group (n = 6); 2-The sham-operated group, which underwent surgery without infarct and received a saline solution in the femoral vein (n = 6; n = 3 sacrificed at 14 d and n = 3 sacrificed at 3 months); 3-The control group, which underwent surgery with permanent middle cerebral artery occlusion (pMCAO) and received the saline infusion in the femoral vein (n = 15; n = 10 sacrificed at 14 d and n = 5 sacrificed at 3 months); 4-The hAD-MSCs group, which underwent pMCAO surgery and received an hAD-MSCs infusion in the femoral vein (n = 15; n = 10 sacrificed at 14 d and n = 5 sacrificed at 3 months); 5- The rAD-MSCs group, which underwent pMCAO surgery and received an rAD-MSCs infusion in the femoral vein (n = 15; n = 10 sacrificed at 14 d and n = 5 sacrificed at 3 months).

**Figure 1:**
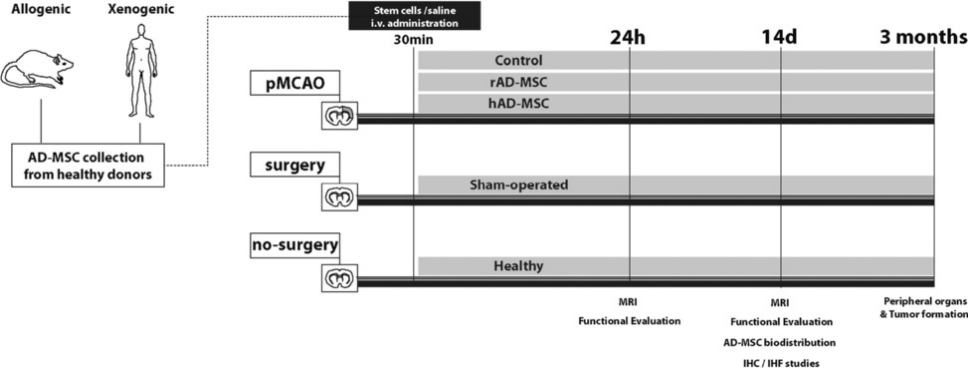
**Experimental protocol.** Animals were subjected to permanent middle cerebral artery occlusion (pMCAO) to induce cerebral ischemia. Rats were randomly divided into 3 groups, which were intravenously injected with saline (control), hAD-MSCs (2 × 10^6^) and rAD-MSCs (2 × 10^6^) in the femoral vein 30 min after stroke. The analyzed parameters were as follows: at 24 h and 14 d, a functional evaluation by Roger’s scale, migration and implantation of MSCs and assessment of lesion size using MRI; at 14 d, migration, implantation and biodistribution of MSCs and a histological and molecular analysis of the peri-infarct zone; at 3 months, tumor formation by inmmunohistochemistry. Sham-operated and healthy animals were also subjected to the same parameter analyses.

Details of the experimental protocol are shown in Figure 1.

#### Surgical procedure

Anesthesia was induced by intraperitoneal injection of a solution of ketamine (25 mg/kg), diazepam (2 mg/kg), and atropine (0.1 mg/kg) at a dose of 2.5 ml/kg. Analgesia was provided by meloxicam 2 mg/kg via a subcutaneous route. A small craniectomy was made above the rhinal fissure over the branch of the right middle cerebral artery (MCA). The MCA branch was permanently ligated just before its bifurcation into the frontal and parietal branches with a 9–0 suture. Both common carotid arteries were then occluded for 60 min as previously described [6,7].

In all animals, the femoral artery was cannulated during surgery and ischemia to allow continuous monitoring of physiological parameters (glycemia, blood gases and blood pressure) (Monitor Omicron ALTEA RGB medical devices).

#### Cell administration

The treatment was administered during the acute phase in order to modulate both protective and repair mechanisms from the very beginning. Thus, intravenous injections of 2×10^6^ AD-MSCs in 650μl saline were administered for 4 min through the femoral vein at 30 min after common carotid artery reperfusion. Control and sham-operated received a saline infusion only. The route, dose and timing of administration were used in a previous study, with good results [2,6].

#### Functional evaluation scale

In all animals, functional evaluations were performed at baseline and at 24 h and 14 d after surgery. Rats were acclimatized in a quiet place for one hour before functional evaluation. A variant of Roger’s functional scale was used to assign scores as follows: 0, no functional deficit; 1, failure to extend forepaw fully; 2, decreased grip of forelimb while tail gently pulled; 3, spontaneous movement in all directions, contralateral circling only if pulled by the tail; 4, circling; 5, walking only when stimulated; 6, unresponsive to stimulation with a depressed level of consciousness; and 7, dead (n = 10 per group) [6,8].

#### Migration and implantation of AD-MSCs as studied by MRI and DiI

For AD-MSCs Endorem labeling, (superparamagnetic iron oxide) we adapted the protocol described by Arbab AS et al. [9]. Cultures were incubated in DMEM and protamine sulphate (6mg/ml) in the presence of Endorem (50μl/ml) at 37°C for 4 h. After incubation, cells were washed with PBS to eliminate excess labeling and were administered to five animals in each group. Migration and implantation were both analyzed using magnetic resonance imaging (MRI) with T2 maps (Flash sequence) at 24 h and 14 d after administration.

For DiI labeling (Celltracker CM-DiI, Invitrogen), cells in suspension were incubated with DiI tracker (1mg/ml) in Hanks (1X) (HBSS, Gibco) for 10 min at 37°C and later, for 30 min at 4°C. After incubation, cells were washed with PBS to eliminate excess labeling and were administered to animals (n = 5 animals per group). Migration and implantation were then analyzed using immunofluorescence at 14 d after administration.

Cell viability was checked by Trypan blue staining, showing a survival >90%. Neither labeling agent affected the baseline characteristics of the MSCs.

#### Biodistribution and tumor formation

Biodistribution of labeled AD-MSCs (2×10^6^) with DiI after IV administration was analyzed using immunofluorescence techniques in treated animals at 14 d after administration. Cryosections (10 μm thick) of brain, kidney, liver, lung and spleen were labeled with 4’,6-diamidino-2-phenylindole (DAPI) and CD11b/c (monoclonal antibody diluted 1:300, BD Biosciences) and were analyzed by immunofluorescence staining.

Moreover, tumor formation and the presence of infiltrating cells were analyzed in the brain and the peripheral organs at three months after AD-MSCs IV administration in five treated animals using gross macroscopic study and hematoxylin and eosin (H&E) staining.

#### Measurement of lesion size by MRI and H&E

Lesion size was analyzed at 24 h and 14 d after surgery on MRI (Bruker Pharmascan Germany 7 Tesla horizontal bore magnets) using T2 maps (RARE 8 T2, 180° flip angle, 3 averages). Ten contiguous coronal slices (thickness: 1 mm) were acquired with a field of view of 35 X 35 mm and a matrix size of 256 X 256 [repetition time (TR) 3000 ms, echo time (TE) 29.5 ms, imaging time 25.5 min, 3 averages]. All images were processed using the J 1.42 Image program (NIH software). To correct for the brain edema effect, lesion size was determined by an indirect method: (infarct area) = (area of the intact contralateral hemisphere)–(area of the intact ipsilateral hemisphere) [2,10].

Lesion size was also estimated with H&E staining of brain sections at 14 d. The samples were coronally sectioned in 10 μm thick slices and digitalized images were made of these slices (Epson Perfection 1260 scanner) and were used to automatically measure the ischemic area (Image Pro plus 4.0, Media Cybernetics, USA) [11]. To check whether H&E showed the infarct area, MAP-2 staining (polyclonal antibody diluted 1:1000, Millipore) and GFAP staining (monoclonal antibody diluted 1:400, Chemicon) was performed.

#### Cell death

Cell death was detected in the peri-infarct zone by TUNEL staining (TdT-FragEL DNA Fragmentation Detection Kit, Oncogene Research Products). Cell counts were performed on one slice from each animal (n = 10 per group), which was taken at 1.6mm posterior to the bregma. The number of positive cells was counted in a minimum of 10 different microscopic fields based on their nuclear morphology and dark color [2] using a 40× objective and image analysis software (Image-Pro Plus 4.1, Media Cybernetics).

#### Immunofluorescence

The peri-infarct area was analyzed in brain sections using various immunofluorescent antibodies to mark: astrocytes with glial fibrillary acid protein (GFAP) (monoclonal antibody diluted 1:400, Chemicon); vascular endothelial growth factor with VEGF (polyclonal antibody diluted 1:500, Millipore), synaptogenesis with synaptophysin (SYP) (monoclonal antibody diluted 1:200, Sigma). Secondary antibodies for immunofluorescence were goat anti-mouse Alexa Fluor 488 and anti-rabbit Alexa Fluor 594 (1: 750, Invitrogen). All sections were mounted with H-1200 VectaShield mounting medium for fluorescence with DAPI (ATOM). Samples were examined using a LEICA TCS SPE spectral confocal microscope (Leica Microsystems, Heidelberg, Germany) and the confocal images were analyzed using LEICA software LAS AF, version 2.0.1 Build 2043. The images were acquired as a confocal maximum projection.

#### Western blot

Proteins were isolated from peri-infarct tissue and their concentrations determined using a BCA protein assay kit (Pierce). Twenty micrograms of protein were loaded onto 10% acrylamide SDS-gels. Following electrophoresis at 100 V for 1 h, the protein was transferred to PVDF membranes (Bio-Rad). Membranes were blocked in 5% fat-free dry milk dissolved in Tris-buffered saline pH 8.0 (TBS) plus 0.1% Tween-20 (TBS-T) for 1 h and probed overnight at 4°C with the following antibodies at the designated dilutions: GFAP (monoclonal antibody diluted 1:400, Chemicon); VEGF (polyclonal antibody diluted 1:500, Millipore); SYP (monoclonal antibody diluted 1:200, Sigma); and β-actin (monoclonal antibody diluted 1:400, Sigma), which was used as a normalizing control. After rinsing with 0.5% TBS-T solution, the membranes were incubated with the secondary antibody, a donkey anti-rabbit and anti-mouse antibody conjugated with horseradish peroxidase (HRP) for 1 h at room temperature. Signals were detected by enhanced chemiluminescence (ECL, Amersham) before exposure on radiographic film. The density of stained bands was scanned and quantified (arbitrary units, AU) by Scion Image and 1-D Manager Version 2.1 (Scion Corporation, Maryland, US).

#### Statistical analysis

Quantitative data are shown as mean values ± SD. The Kruskal–Wallis test followed by the Mann–Whitney test were used to compare the functional evaluation score, lesion size and cell death, while VEGF, SYP and GFAP levels were compared among the various groups. Values of p < 0.05 were considered significant at a 95% confidence level (using statistical software SPSS 16 for Windows).

## Results

### Characterization of AD-MSCs

**Figure 2:**
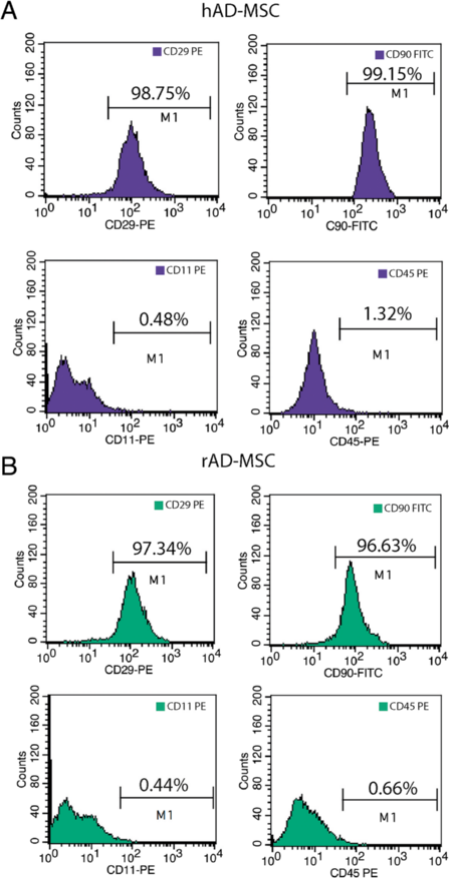
**Characterization of adipose tissue-derived mesenchymal stem cells.** The levels of CD90-FITC, CD29-PE, CD45-PE and CD11b-PE markers were analyzed by flow cytometry in hAD-MSCs **(A)** and rAD-MSCs **(B)**.

We confirmed that CD90-FITC (AbD Serotec) and CD29-PE (AbD Serotec) surface markers were positive (all >90%) in AD-MSCs whereas the CD45-PE (AbD Serotec) and CD11b-PE (AbD Serotec) surface markers were negative (all <3%) (Figure 2). The AD-MSCs showed typical, fibroblast-like cell morphology.

### Xenogeneic and allogeneic AD-MSCs administration improves functional recovery after pMCAO

**Figure 3:**
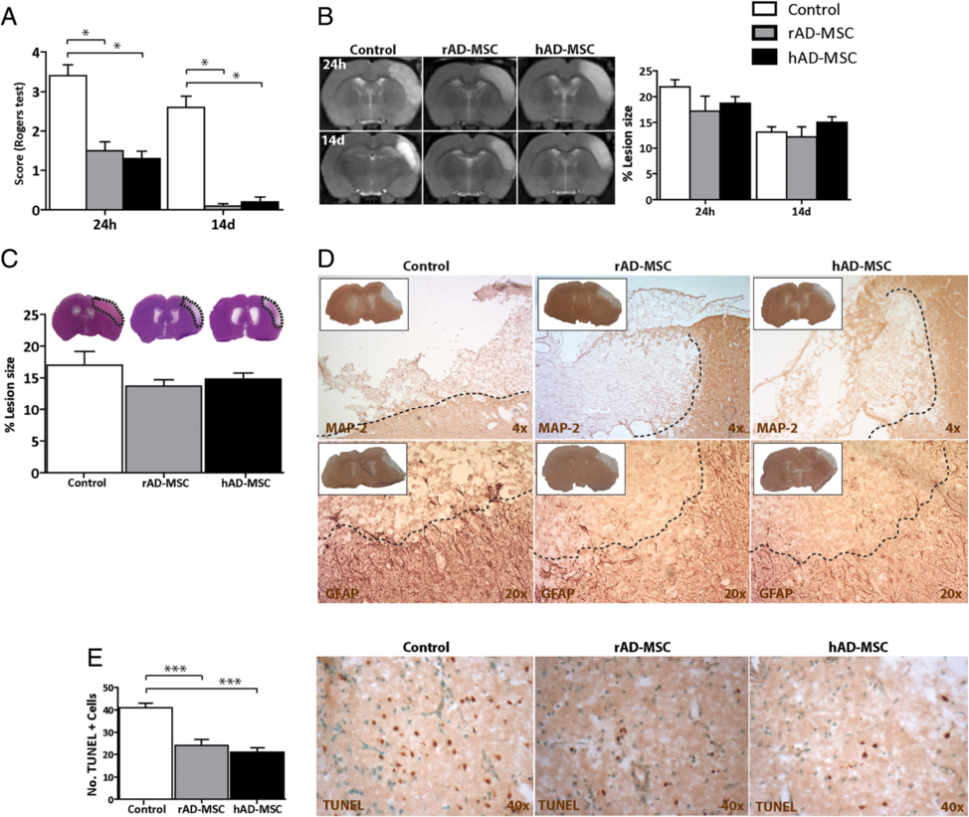
**Effects of AD-MSCs on functional recovery, lesion size and cell death after pMCAO**. Functional outcome was measured by Roger’s test after pMCAO **(A)**. Lesion size was measured using in vivo MRI at 24 h and 14 d **(B)** and H&E at 14 d **(C)**. MAP-2 (4×) and GFAP (20×) staining was used to confirm the lesion zone **(D)**. Cell death was evaluated by TUNEL staining in the peri-infarct zone at 14 d (40×) **(E)** (scale bars = 20 μm). Results are presented as mean ± SD (n = 10 per group) (p < 0.05).

Animals were functionally evaluated at 24 h and 14 d and the corresponding score was obtained for each group (Figure 3A). The sham-operated group did not show functional deficits. The treated groups showed good functional recovery without significant differences between treated groups at either time point. In the Rogers’ test, the hAD-MSCs (1.3 ± 0.6;0.2 ± 0.4) and rAD-MSCs (1.5 ± 0.7;0 ± 0) groups had significantly improved functional recovery compared with the control group (3.4 ± 0.89; 2.6 ± 0.89) at 24 h and at 14 d, respectively (p < 0.05).

### Xenogeneic and allogeneic AD-MSCs administration does not reduce infarct size after pMCAO

Lesion sizes at 24 h and 14 d after the experimental procedure were evaluated on MRI (Figure 3B). Neither of the treatments, hAD-MSCs (18.7 ± 4.2; 15.01 ± 3.4) or rAD-MSCs (17.2 ± 9.2; 12.2 ± 6), reduced the lesion size significantly with respect to the control group (21.89 ± 4.38; 13.12 ± 3.16). In addition, H&E staining showed that neither hAD-MSCs (14.8 ± 3.06) or rAD-MSCs (13.7 ± 3.20), had decreased lesion size significantly with respect to the control group (16.98 ± 7.03) at 14 d (Figure 3C). These results were confirmed by MAP-2 and GFAP staining (Figure 3D).

### Xenogeneic and allogeneic AD-MSCs administration decreased cell death after pMCAO

Sham-operated animals did not show TUNEL+ cells. There were no TUNEL+ cells in the contralateral hemisphere in any operated animal. At 14 d, the control group (41 ± 6.4) showed significantly more TUNEL+ cells than the hAD-MSCs (21 ± 6.7) and rAD-MSCs (24 ± 8.8) groups in the peri-infarct zone (Figure 3E).

### Xenogeneic and allogeneic AD-MSCs administration modified brain marker levels after pMCAO

**Figure 4:**
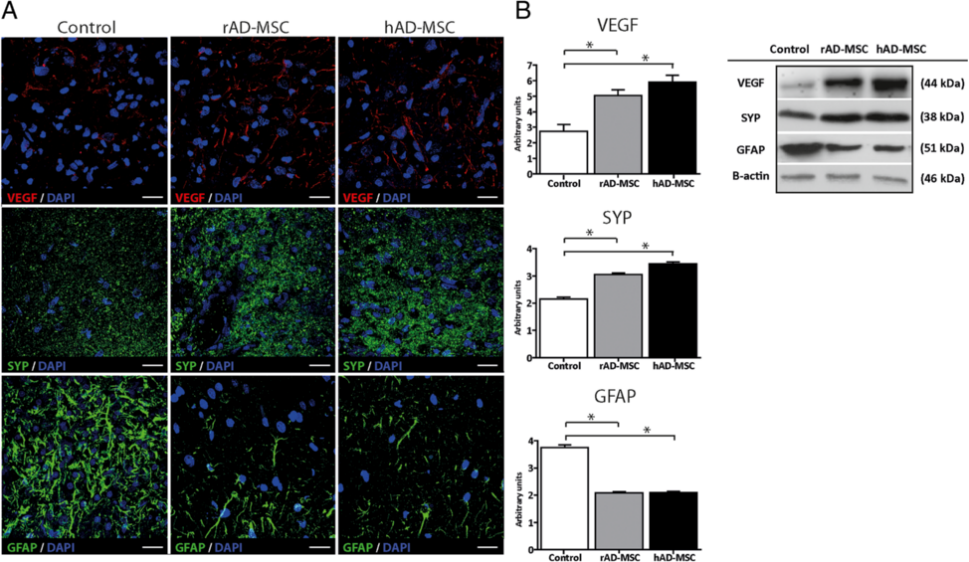
**Expression of brain markers in the peri-infarct zone at 14 d after pMCAO.** Levels of vascular endothelial growth factor (VEGF), synaptophysin (SYP) and glial fibrillary acid protein (GFAP) were analyzed in the peri-infarct zone (scale bars = 20 μm) using immunofluorescence **(A)** and Western blot **(B)**. Results are presented as mean ± SD (n = 3 per group) (p < 0.05).

At 14 d after pMCAO, the levels of brain repair markers (VEGF and SYP) were analyzed by immunofluorescence (Figure 4A) and confirmed with the Western blot technique (Figure 4B). Compared with the control group (2.75 ± 1.38 AU), rat brain VEGF levels were significantly higher after administration of either hAD-MSCs (5.92 ± 1.38 AU) or rAD-MSCs (5.05 ± 1.16 AU). The levels of SYP were also significantly higher than in the control group (2.15 ± 0.22 AU) after hAD-MSCs (3.45 ± 0.23 AU) and rAD-MSCs (3.06 ± 0.15 AU) treatments. Regarding the astrocyte marker GFAP, its levels were significantly decreased after treatment with either hAD-MSCs (2.098 ± 0.13 AU) or rAD-MSCs (2.075 ± 0.17 AU) in comparison with the control group (3.76 ± 0.31 AU). In no case did we observe any significant differences in brain markers between the hAD-MSCs- or rAD-MSCs-treated groups.

### Neither AD-MSCs migration nor implantation was observed in the injured brain area after IV administration

**Figure 5:**
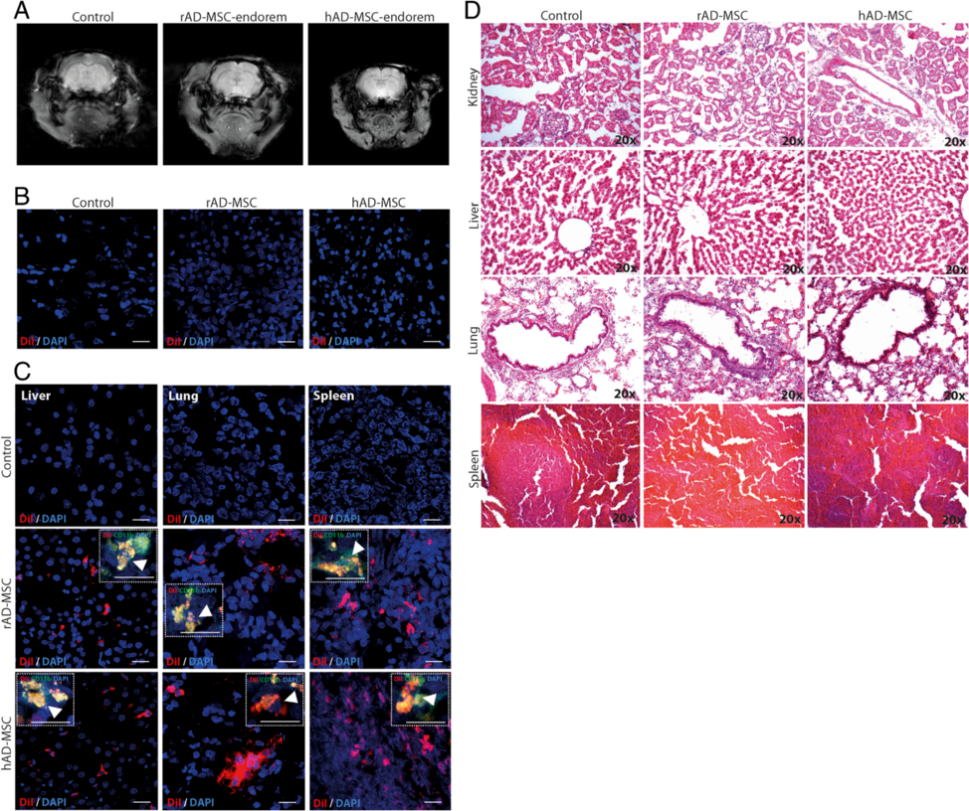
**AD-MSCs biodistribution and histological study of peripheral organs. A, B)** Migration and implantation of AD-MSCs after IV injection were studied in the ischemic brain by either MRI or immunofluorescence at 14 d. **C)** The presence of AD-MSCs administered to treated groups was checked in peripheral organs: liver, lungs and spleen at 14 d by DiI staining and also by co-labeling with CD11b/c (detail). **D)** H&E staining was used to study possible tumor formation in the kidney, liver, lung or spleen at 3 months after AD-MSCs administration (scale bars = 20 μm) (n = 5 per group).

The possible migration and implantation of AD-MSCs were studied by MRI at 24 h and 14 d and by immunofluorescence in DiI labeled cells at 14 d. Labeled AD-MSCs were not observed in the control group because they were not administered to this group. Endorem and DiI-labeled AD-MSCs were only injected into the treated groups. However, neither migration nor implantation was observed on either MRI or immunofluorescence images in the injured brain area after IV administration of either hAD-MSCs or rAD-MSCs (Figure 5A,B). Nevertheless, we did observe AD-MSCs biodistribution into peripheral organs: the liver, lung and spleen. We found DiI co-labeling with CD11b, indicating that the administered stem cells were incorporated into macrophages. However, we did not observe differences between the rAD-MSCs and hAD-MSCs groups (Figure 5C).

### Administration of xenogeneic or allogeneic AD-MSCs was safe after pMCAO

The administration of hAD-MSCs or rAD-MSCs showed no side effects or tumor formation at three months in any treatment group, demonstrating the safety of this treatment. When we analyzed histological sections of various organs where we found labeled AD-MSCs (spleen, lung and liver) at three months after administration, no tumor formation or cell infiltrate were observed. Moreover, other organs, such as the kidney, showed no tissue alterations. No differences were observed when comparing rAD-MSCs and hAD-MSCs (xenogeneic and allogeneic, respectively) (Figure 5D).

## Discussion

In our study, the IV administration of MSCs from human adipose tissue (xenogeneic administration) was shown to be as safe as those from rat adipose tissue (allogeneic administration) in an experimental stroke model, producing no critical adverse effects or evidence of tumor formation during the three-month follow-up. Furthermore, the treatment was effective, inducing decreased cell death and increased brain plasticity markers.

MSCs have shown great promise as a cell-based therapy for many human diseases such as stroke, as a result of their multipotency, high proliferation capacity and low immunogenicity, including immunomodulatory protective and repair effects. Moreover, these types of cells are known to secrete multiple growth factors and thereby have cytoprotective effects in various injury models. There are several MSCs sources, including umbilical cord blood, bone marrow and adipose tissue. All of these sources have been used in experimental animals and some have been used in clinical trials such as NCT00875654, NCT01297413, NCT02283879 [12].

Among all of these sources, AD-MSCs are abundant, have greater availability, are ethically acceptable and are easy to obtain without invasive surgery. They can be obtained in high amounts through simple procedures such as liposuction or abdominoplasty and they have been used in a number of animal models of stroke, in which improved functional scores, reductions in infarct size and modulation of inflammation were observed [4,13]. Moreover, AD-MSCs have low MHC-I expression and lack MHC-II molecules, suggesting that they are not likely induce rejection [14]. Along these lines, previous studies from our group showed no symptoms of rejection after acute allogeneic administration of MSCs after pMCAO in rats [2,6]. Therefore, AD-MSCs are especially interesting for clinical application to stroke [15-17], and in fact, phase I and II clinical trials are now ongoing to evaluate the safety of AD-MSCs administration for other pathologies such as NCT01300598, NCT01649700, NCT01739504 [12].

### Xenogeneic and allogeneic AD-MSCs administration improve functional recovery after pMCAO

In experimental animal models, administration of human or rat MSCs has shown a significant effect on functional outcome in stroke [2,6,13,18-21]. Our present results have shown that hAD-MSCs as well as rAD-MSCs administration is effective after cerebral ischemia in terms of improving functional recovery. Both treatments showed the same benefit regardless of cell origin (xenogeneic vs. allogeneic). Because only one motor functional evaluation test was used, futures studies should analyze the effects of AD-MSCs administration on coordination, cognitive and memory impairments after stroke.

### Neither AD-MSCs migration nor implantation was observed in the injured brain area after IV administration

Previous studies showed good results in terms of functional outcomes after IV MSCs administration in the absence of cell grafting in the lesion area [6]. In the present study, although functional outcomes were good, neither IV-administered hAD-MSCs nor AD-MSCs migrated or showed implantation in the injured brain, suggesting that it might not be necessary for the AD-MSCs reach the brain in order to exert their therapeutic effects. In fact, we observed that after IV administration, MSCs were found in various peripheral organs such as the spleen, lung and liver, in agreement with previous studies in which the authors hypothesized that the administered cells might act indirectly from peripheral organs (spleen, lung and liver) [22-24].

Regarding cell clearance from peripheral organs, a recent study suggested that xenografting has an impact on cell clearance from the brain and faster relocation of human MSCs to the liver and spleen compared to rat MSCs [25]. However, in our study we observed no difference either in implantation or clearance between rAD-MSCs and hAD-MSCs, as measured with co-labeling with macrophage staining.

In a translational context, safety and tumor formation should be analyzed for stem cell therapy optimization. Despite earlier concerns regarding the potential tumorigenic property of stem cells, a survey of current studies reveals only a few instances of tumor formation following MSCs administration [26]. One study showed that xenotransplantation of human MSCs into immunocompetent wild-type rats did not result in any of the apparent gross or microscopic findings consistent with abnormal immunologic reactions [27]. In our study, AD-MSCs demonstrated safety and no adverse effects independent of cellular origin (xenogeneic or allogeneic source), and they did not cause tumor formation in any organ at three months after administration.

### Xenogeneic and allogeneic AD-MSCs administration decreased cell death but not lesion size after pMCAO

In a previous study, AD-MSCs markedly attenuated brain infarct size in rats [7]. However, in the present study, although an improvement functional recovery was observed even at 24 h, AD-MSCs administration did not reduce lesion sizes in treated animals in comparison to control animals. However, this apparent lack of protection was compensated by a reduction in cell death, as we found that the number of TUNEL-positive cells was significantly higher in the control group than in MSCs-treated groups. These results suggest that although the infarct size is not reduced, early tissue preservation occurs as there is reduced cell death. This result agrees with previous studies in which AD-MSCs also inhibited cell death after stroke [2,4,28].

### Xenogeneic and allogeneic AD-MSCs administration modified brain marker levels after pMCAO

Numerous studies have shown that MSCs might exert their beneficial effects through secreted trophic factors, which directly or indirectly promote ischemic brain tissue repair. MSCs are stimulated to secrete various trophic factors, including brain-derived neurotrophic factor (BDNF), VEGF, nerve growth factor (NGF) and HGF, which have been implicated in endogenous repair mechanisms [19].

In line with these studies, at 14 d, we found that the animals receiving AD-MSCs treatment showed a significant increase in levels of VEGF and SYP and a significant decrease in GFAP markers. Administered MSCs might play roles in angiogenesis (VEGF) and synaptogenesis (SYP), processes implicated in brain repair. Other studies have reported similar results after MSCs administration with an increase in VEGF [3,6,29,30] as well as in SYP markers [2,31]. In respect to GFAP, an astrocyte marker, in our study we observed a decrease in its expression in the peri-infarct zone in treated animals with respect to the control group, which is in line with a previous report by Leu et al. [4]. Brain damage triggers reactive gliosis, which is characterized by increased expression of specific markers such as GFAP surrounding the injury site. In the reactive process, the astrocytes participate in glial scar formation, becoming hypertrophied and inhibiting axonal regeneration [32].

The beneficial effects after MSCs administration could be partly mediated by paracrine factors produced by these administered cells; however mechanisms of action for this mediation are as yet unknown. The mechanisms would likely involve stimulation of an active repair response that becomes operational in the acute to subacute time frame [22].

## Conclusions

The administration of hAD-MSCs or rAD-MSCs has been shown to be safe and without side effects or tumor formation. Furthermore, both treatment groups showed equal efficacy in terms of functional recovery and decreased ischemic brain damage (reduction in cell death and glial scarring) as well as increases in angiogenesis and synaptogenesis markers.

## References

[1] World Health Organization. The top 10 causes of death – Homepage. [http://www.who.int/mediacentre/factsheets/fs310/en/]

[2] Gutiérrez-Fernández M, Rodríguez-Frutos B, Ramos-Cejudo J, Vallejo-Cremades MT, Fuentes B, Cerdan S (2013). Effects of intravenous administration of allogenic bone marrow- and adipose tissue-derived mesenchymal stem cells on functional recovery and brain repair markers in experimental ischemic stroke. Stem Cell Res Ther.

[3] Ikegame Y, Yamashita K, Hayashi S, Mizuno H, Tawada M, You F (2011). Comparison of mesenchymal stem cells from adipose tissue and bone marrow for ischemic stroke therapy. Cytotherapy.

[4] Leu S, Lin YC, Yuen CM, Yen CH, Kao YH, Sun CK (2010). Adipose-derived mesenchymal stem cells markedly attenuate brain infarct size and improve neurological function in rats. J Transl Med.

[5] Zhang HT, Liu ZL, Yao XQ, Yang ZJ, Xu RX (2012). Neural differentiation ability of mesenchymal stromal cells from bone marrow and adipose tissue: a comparative study. Cytotherapy.

[6] Gutiérrez-Fernández M, Rodríguez-Frutos B, Álvarez-Grech J, Vallejo-Cremades MT, Expósito-Alcaide M, Merino J (2011). Functional recovery after hematic administration of allogenic mesenchymal stem cells in acute ischemic stroke in rats. Neuroscience.

[7] Ramos-Cejudo J, Gutiérrez-Fernández M, Rodríguez-Frutos B, Expósito-Alcaide M, Sánchez-Cabo F, Dopazo A (2012). Spatial and temporal gene expression differences in core and periinfarct areas in experimental stroke: a microarray analysis. PLoS One.

[8] Rogers DC, Campbell CA, Stretton JL, Mackay KB (1997). Correlation between motor impairment and infarct volume after permanent and transient middle cerebral artery occlusion in the rat. Stroke.

[9] Arbab AS, Yocum GT, Kalish H, Jordan EK, Anderson SA, Khakoo AY (2004). Efficient magnetic cell labeling with protamine sulfate complexed to ferumoxides for cellular MRI. Blood.

[10] Swanson RA, Morton MT, Tsao-Wu G, Savalos RA, Davidson C, Sharp FR (1990). A semiautomated method for measuring brain infarct volume. J Cereb Blood Flow Metab.

[11] Avendaño C, Roda JM, Carceller F, Díez-Tejedor E (1995). Morphometric study of focal cerebral ischemia in rats: a stereological evaluation. Brain Res.

[12] ClinicalTrials.gov, U.S. National Library of Medicine – Homepage. [www.clinicaltrials.gov]

[13] Kim JM, Lee ST, Chu K, Jung KH, Song EC, Kim SJ (2007). Systemic transplantation of human adipose stem cells attenuated cerebral inflammation and degeneration in a hemorrhagic stroke model. Brain Res.

[14] Javazon EH, Beggs KJ, Flake AW (2004). Mesenchymal stem cells: paradoxes of passaging. Exp Hematol.

[15] Gutiérrez-Fernández M, Fuentes B, Rodríguez-Frutos B, Ramos-Cejudo J, Vallejo-Cremades MT, Díez-Tejedor E (2012). Trophic factors and cell therapy to stimulate brain repair after ischaemic stroke. J Cell Mol Med.

[16] Gutiérrez-Fernández M, Rodríguez-Frutos B, Otero-Ortega L, Ramos-Cejudo J, Fuentes B, Díez-Tejedor E (2013). Adipose tissue-derived stem cells in stroke treatment: from bench to bedside. Discov Med.

[17] Gutiérrez-Fernández M, Rodríguez-Frutos B, Ramos-Cejudo J, Otero-Ortega L, Fuentes B, Díez-Tejedor E (2013). Stem cells for brain repair and recovery after stroke. Expert Opin Biol Ther.

[18] Yang M, Wei X, Li J, Heine LA, Rosenwasser R, Iacovitti L (2010). Changes in host blood factors and brain glia accompanying the functional recovery after systemic administration of bone marrow stem cells in ischemic stroke rats. Cell Transplant.

[19] Hao L, Zou Z, Tian H, Zhang Y, Zhou H, Liu L (2014). Stem cell-based therapies for Ischemic stroke. Biomed Res Int.

[20] Ishizaka S, Horie N, Satoh K, Fukuda Y, Nishida N, Nagata I (2013). Intra-arterial cell transplantation provides timing-dependent cell distribution and functional recovery after stroke. Stroke.

[21] Otero L, Zurita M, Bonilla C, Aguayo C, Rico MA, Rodríguez A (2012). Allogeneic bone marrow stromal cell transplantation after cerebral hemorrhage achieves cell transdifferentiation and modulates endogenous neurogenesis. Cytotherapy.

[22] Savitz SI, Cramer SC, Wechsler L (2014). Stem cells as an emerging paradigm in stroke 3: enhancing the development of clinical trials. Stroke.

[23] Pendharkar AV, Chua JY, Andres RH, Wang N, Gaeta X, Wang H (2010). Biodistribution of neural stem cells after intravascular therapy for hypoxic-ischemia. Stroke.

[24] Lappalainen RS, Narkilahti S, Huhtala T, Liimatainen T, Suuronen T, Narvanen A (2008). The SPECT imaging shows the accumulation of neural progenitor cells into internal organs after systemic administration in middle cerebral artery occlusion rats. Neurosci Lett.

[25] Khabbal J, Kerkelä E, Mitkari B, Raki M, Nystedt J, Mikkonen V, et al. Differential clearance of rat and human bone marrow-derived mesenchymal stem cells from the brain after intra-arterial infusion in rats. Cell Transplant. 2014; doi:10.3727/096368914X679336. 10.3727/096368914X679336 24593908

[26] Tolar J, Nauta AJ, Osborn MJ, Panoskaltsis Mortari A, McElmurry RT, Bell S (2007). Sarcoma derived from cultured mesenchymal stem cells. Stem Cells.

[27] Ahn SY, Chang YS, Sung DK, Sung SI, Yoo HS, Lee JH (2013). Mesenchymal stem cells prevent hydrocephalus after severe intraventricular hemorrhage. Stroke.

[28] Yang YC, Liu BS, Shen CC, Lin CH, Chiao MT, Cheng HC (2011). Transplantation of adipose tissue-derived stem cells for treatment of focal cerebral ischemia. Curr Neurovasc Res.

[29] Wakabayashi K, Nagai A, Sheikh AM, Shiota Y, Narantuya D, Watanabe T (2010). Transplantation of human mesenchymal stem cells promotes functional improvement and increased expression of neurotrophic factors in a rat focal cerebral ischemia model. J Neurosci Res.

[30] Guo F, Lv S, Lou Y, Tu W, Liao W, Wang Y (2012). Bone marrow stromal cells enhance the angiogenesis in ischaemic cortex after stroke: involvement of notch signalling. Cell Biol Int.

[31] Stroemer RP, Kent TA, Hulsebosch CE (1995). Neocortical neural sprouting, synaptogenesis, and behavioral recovery after neocortical infarction in rats. Stroke.

[32] Sofroniew MV (2005). Reactive astrocytes in neural repair and protection. Neuroscientist.

